# *Quorum-Quenching* Activity of *Myrtus communis* Corsican Essential Oil Against the Marine Bacterium *Aliivibrio fischeri*

**DOI:** 10.3390/microorganisms13061325

**Published:** 2025-06-06

**Authors:** Elisa Hardy, Jean-Pierre Poli, Ange Bighelli, Mathieu Paoli, Thomas Maroselli, Liliane Berti, Elodie Guinoiseau

**Affiliations:** 1 CNRS, Axe Environnement et Santé, Projet Ressources Naturelles, UMR 6134, SPE, Université de Corse, Campus Grimaldi BP 52, 20250 Corte, France; hardy_e@univ-corse.fr (E.H.); berti_l@univ-corse.fr (L.B.); guinoiseau_e@univ-corse.fr (E.G.); 2 CNRS, Axe Phytochimie et Interactions, Projet Ressources Naturelles, UMR 6134, SPE, Université de Corse, Route des Sanguinaires, 20000 Ajaccio, France; bighelli_a@univ-corse.fr (A.B.); paoli_m@univ-corse.fr (M.P.); maroselli_t@univ-corse.fr (T.M.)

**Keywords:** *Aliivibrio fischeri*, essential oils, biofilm, bioluminescence, minor compounds

## Abstract

The *quorum-quenching* activity of essential oils (EOs) from Corsican aromatic plants was evaluated using the marine bacterium *Aliivibrio fischeri* as a model system. Among the eleven EOs screened, *Myrtus communis* EO showed significant interference with QS-regulated phenotypes (swimming motility, bioluminescence, and biofilm formation). Its activity was compared to *Origanum vulgaris* EO, known for its high carvacrol content and potent QS inhibition. The fractionation of *M. communis* EO revealed that its most polar fractions exhibited comparable levels of QS-disrupting activity. These chromatographic fractions significantly affected QS-controlled traits, indicating that minor or less volatile compounds may contribute to, or enhance, the overall bioactivity. Furthermore, *M. communis* EO and its polar fractions displayed stronger anti-QS effects against *A. fischeri* than *O. vulgaris* EO. These results highlight *M. communis* EO as a promising source of natural QS inhibitors and underscore the importance of exploring both complete EOs and their active fractions. This study supports the valorization of Mediterranean endemic flora as a reservoir of bioactive compounds, tested on a model system *A. fischeri*, and encourages future research on the potential of *Myrtus communis* against clinical bacterial isolates and the development of novel anti-virulence strategies.

## 1. Introduction

The emergence and accumulation of bacterial resistance limit the effectiveness of certain antibiotic treatments. The selection pressure exerted by antibiotic molecules to kill bacteria not only increases resistance but also promotes bacterial exchange. To fight bacterial resistance, one area of research focuses on disrupting bacterial communication. This mechanism, known as *quorum*-*sensing* (QS), plays a crucial role in bacterial organization, resistance and virulence. A key element of QS is a family of specific molecules called autoinducers. By competing with or inhibiting autoinducers, the QS mechanism can be disrupted through *quorum*-*quenching* activity [1,2]. To study this activity in vitro, specific bacterial models are used, such as *Chromobacterium violaceum* (a purple-pigmented bacterium) or *Aliivibrio fischeri* (a bioluminescent bacterium), the latter is the first bacteria in which the QS mechanism was described [3,4,5,6]. This marine symbiotic bacterium of the squid *Euprymna scolopes* is a Gram-negative bacteria capable of producing lophotrichous flagellar tufts, biofilm, and blue-green luminescence through QS regulation [7,8].

The *Vibrionaceae* family includes another luminescent bacterium, *Vibrio harveyi*, the toxin-producing pathogenic bacterium *Vibrio cholerae*, and a group of opportunistic pathogens that affect both shellfish and humans, including *Vibrio parahaemolyticus*, *Vibrio alginolyticus*, *Vibrio anguillarum*, and *Vibrio vulnificus* [9,10,11]. All of these bacteria are implicated in gastroenteric infections following shellfish consumption, with *Vibrio vulnificus* also known as the flesh-eating bacterium. *Vibrio vulnificus*, like many species of *Vibrio*, can survive in harsh environments or within the human body, with flagellar development and biofilm formation providing dispersal and protective abilities.

*Aliivibrio fischeri* is a model to test potentials *quorum-quenching* activities of natural products such as essential oils, aiming to limit bacterial proliferation, such as biofilm formation on coastal surfaces, ship hull, or abiotic surface (biofouling) and mobility to development sites, which can lead to infections all without increasing antibacterial resistance [7,8,12].

Natural extracts are well known for their biological activities across various fields of application such as cosmetics and pharmaceuticals. Highly concentrated active molecules found in essential oils (EOs) from leaves and flowers, or in honey from bee pollen [13], are also used as preservatives in cosmetic and food products [14,15,16]. Essential oils are complex mixtures of volatile chemical compounds that have been extensively studied for their wide range of biological activities, including antibacterial, antifungal, and antioxidant effects. The antimicrobial activity of EOs results from additive or synergistic interactions between different molecules, which are expressed or extracted through steam distillation, hydrodistillation, or cold pressing. The type of plant, cultivation method, period, and geography directly influence the complexity of essential oil composition [17,18,19]. EOs consist of organic volatile compounds, generally mono- and sesquiterpenes as well as phenylpropanoids, with various functional groups (alcohols, ketones, aldehydes, esters, oxides, etc.) [20]. Monoterpenes are widely represented in EOs and include different functional groups. For example, geraniol, linalool, and carvacrol are monoterpene alcohols, while citral (a combination of geranial and neral) is a monoterpene aldehyde. Other compounds, like limonene, *p*-cymene, and α-pinene are monoterpene hydrocarbons. The abundance of these compounds varies depending on the plant family, such as monoterpenes from evergreens [21]. The recurring composition of certain plant species, such as *Origanum* spp. or *Thymus* spp., with carvacrol or thymol as active molecules, differs only in the ratio of these molecules [22,23,24].

The direct antibacterial activity of EOs, such as *Origanum vulgaris* or *Melaleuca alternifolia*, including their ability to disrupt membrane integrity or deplete adenosine triphosphate concentration, is well documented [25,26,27]. EOs are among the preferred mixtures used to study synergistic interactions, such as carvacrol and *p*-cymene, which demonstrate activity against the foodborne pathogen *Vibrio cholerae* [28]. EOs from the Mediterranean, particularly those from Corsica such as *Inula graveolens* or *Cistus ladaniferus*, have demonstrated antibacterial activity against *Staphylococcus* or *Enterobacter* [29,30]. Some, like *Mentha suaveolens* ssp. *insularis*, have shown strong *quorum-quenching* activity due to *cis-cis*-*p*-menthenolide, one of the main compounds in the oil, which inhibits biofilm formation and violacein production in *Chromobacterium violaceum* [31]. Some others, like oregano essential oil and its carvacrol-concentrated fractions, have demonstrated strong potential to reduce the motility of several bacteria associated with urinary tract infections [32]. In most cases, the major compounds of EOs are responsible for the antibacterial activities due to their proportion in the entire extract, but sometimes minor compounds, through synergistic interactions or strong individual activities, may play a crucial role [33].

In this study, the effectiveness of eleven Corsican EOs was evaluated on three mechanisms regulated by the QS system, namely bioluminescence, motility, and biofilm formation, in *Aliivibrio fischeri* and compared to a carvacrol chemotype *Origanum vulgaris* EO used as internal control. The EO showing the highest activity for each of the three tested parameters, *Myrtus communis*, was selected and then fractionated by chromatography to investigate the chemical families’ molecules involved in the anti-QS activity observed.

## 2. Materials and Methods

### 2.1. Bioactive Substances

Twelve essential oils were evaluated, including eleven sourced from Corsica and one non-island reference oil, *Origanum vulgaris*, which is highly concentrated in carvacrol. This reference oil was purchased from the French producer Lumiflor (Aubagne, France) and distilled from the aerial parts of the plant. Two Corsican essential oil producers were selected: Astratella (Lumio, France) and Amuredda (Prunelli-di-Fiumorbu, France). Five of the eleven Corsican essential oils were provided by Astratella, namely *Eucalyptus polybractea*, *Eucalyptus globulus*, *Rosmarinus officinalis*, and *Pistacia lentiscus*, all distilled from leafy branches. Astratella also supplies the essential oil extracted from the aerial parts of *Inula graveolens*. The remaining six essential oils were obtained from Amuredda, namely *Lippia citriodora*, *Myrtus communis*, *Pelargonium asperum*, *Mentha piperita*, and *Lavandula hybrida*, which were all distilled from leaves, except for *Cymbopogon winterianus*, which was distilled from the plant’s aerial parts.

### 2.2. Column Chromatography of Myrtus communis EO

The *Myrtus communis* leaves essential oil (*M. communis* EO; 2.563 g) were chromatographed on silica gel (63–200 µm, 60 Å, 53 g) with a gradient of solvent (pentane/diethyl ether from 100/0 to 0/100), yielding four fractions (F1–F4): F1 (100/0; 1.246 g), F2 (98/2; 664.2 mg), F3 (90/10; 53.0 mg), and F4 (0/100; 453.1 mg).

### 2.3. GC-FID Analysis

GC-FID analyses were carried out using a Clarus 500 Perkin Elmer (Perkin Elmer, Courtaboeuf, France) system equipped with an FID and two fused-silica capillary columns (50 m × 0.22 mm, film thickness 0.25 µm), BP-1 (polydimethylsiloxane), and BP-20 (polyethylene glycol). The oven temperature was programmed from 60 °C to 220 °C at 2 °C/min and then held isothermal at 220 °C for 20 min; injector temperature: 250 °C; detector temperature: 250 °C; carrier gas: H_2_ (0.8 mL/min); split: 1/60; injected volume: 0.5 µL. The relative proportions of the essential oil constituents were expressed as percentages obtained by peak-area normalization, without using correction factors. Retention indices (RI) were determined relative to the retention times of a series of n-alkanes with linear interpolation (Target Compounds software from Perkin Elmer).

### 2.4. GC-MS Analysis

GC-MS analyses were performed on a Clarus SQ8S Perkin Elmer TurboMass detector (quadrupole), directly coupled to a Clarus 580 Perkin-Elmer Autosystem XL, equipped with a BP-1 (polydimethylsiloxane) fused-silica capillary column (60 m × 0.22 mm i.d., film thickness 0.25 µm). The oven temperature was programmed from 60 °C to 230 °C at 2°/min and then held isothermally at 230 °C for 45 min; injector temp., 250 °C; ion-source temp., 150 °C; carrier gas, He (1 mL/min); split ratio, 1:80; injection volume, 0.2 µL; ionization energy, 70 eV. The electron ionization (EI) mass spectra were acquired from within the mass range of 35–350 Da.

### 2.5. Identification of Individual Components

Essential oil samples and fractions of chromatography were submitted to GC, in combination with retention indices (RI) and GC-MS (50 mg of essential oil or fraction CC diluted in 500 µL of CHCl_3_). Identification of the individual components was carried out as follows: (i) by the comparison of their GC retention indices (RI) on polar and apolar columns with those of reference compounds compiled in a laboratory-built library and with literature data [34,35,36]; (ii) via computer matching against commercial mass spectral libraries [37].

### 2.6. Bacterial Strains and Growth Conditions

The bacterial strain *Aliivibrio fischeri* (formerly *Vibrio fischeri*) was obtained from the Collection Institut Pasteur (CIP 103206T) and cultivated according to an adaptation of the Christensen and Visick protocol [23] at temperatures between 25 and 28 °C in SWTO broth.

### 2.7. Minimal Inhibitory Concentration Test

The MIC determination was performed in microplates following the Guinoiseau et al. protocol with some adaptations [30]. All essential oils were solubilized in absolute ethanol (VWR, Radnor, PA, USA). Positive growth controls were performed using absolute ethanol in bacterial culture broth, while negative controls consisted of essential oil diluted in sterile culture medium without bacteria. All test solutions were prepared at a final concentration of 1% *v*/*v*, corresponding to 2 µL of absolute ethanol or essential oil diluted in 200 µL of culture broth, with or without bacteria. This yielded a final concentration range from 10,000 ppm to 5 ppm. The minimum inhibitory concentration (MIC) determination enabled the identification of a common sub-inhibitory concentration suitable for all essential oils, based on optical density measurements at 600 nm after 24 h of incubation with shaking.

### 2.8. Swimming Mobility Test

According to the Christensen and Visick protocol [38], SWTO broth was supplemented with 1.25% agar to evaluate swimming and luminescence in semi-liquid media. In accordance with the MIC testing protocol, each well of the microplate was inoculated with 2 µL of essential oil diluted in 200 µL of bacterial broth culture at concentrations between 1 and 2 × 10^8^ CFU·mL^−1^, yielding a final essential oil concentration of 50 ppm. A 5 µL mixture of bacteria and essential oil was plated in the center of the plate using a center shot. The swimming interval was 6 h (±1 h), with four measurements taken starting approximately 6 h post-inoculation. Bacterial swimming was assessed from inoculation to 24 h of incubation at 28 °C, every 6 h.

### 2.9. Bioluminescence Perturbation Assay

Luminescence quantification was performed using a Tecan Infinite M Plex (Tecan, Männedorf, Austria) luminometer. The *inoculum* used for the swimming motility assay was also used for the luminescence kinetics study. The luminescence intervals were set at 10 min throughout a period of 2 h and 30 min at 28 °C. A total of 200 µL of bacterial *inoculum* were inoculated into each well with 2 µL of diluted essential oils. All tests were performed using white (Nunc, Roskilde, Denmark) and transparent (Greiner, Kremsmünster, Austria) plates to control for OD600 measurements.

### 2.10. Relative Antibiofilm Quantification

Relative biofilm quantification was performed using the same *inoculum* as for luminescence after 2 h and 30 min of incubation under the same conditions as described above. After the incubation period, the *inoculum* was removed, and the microplate was washed three times with phosphate buffer. Once the biofilm was dry, 250 µL of crystal violet (Sigma, St. Louis, MO, USA) solution (0.1%) was added to each well and removed after 5 min. After obtaining clear wash water, 250 µL of 20% ethanol/1% trichloroacetic acid was added to each well, and after color homogenization, the absorbance was read at 595 nm.

### 2.11. Statistical Analyses

All data were analyzed using the R 3.6.0 and RStudio 4.3.1 statistical software (http://www.R-project.org, accessed on 20 July 2022) and Rstudio (Rstudio Inc., Boston, MA, USA, version 1, April 1717). All data were expressed as mean values (±standard error) and submitted to two-way ANOVA with an LSD test when a significant difference was detected at *p* < 0.001.

## 3. Results

### 3.1. Selection of Essential Oils and Identification of Their Major Compounds

The twelve EOs were analyzed by GC(RI) and GC-MS, and the major compounds are listed in Table 1. The composition of EOs of Eucalyptus is dominated by 1,8-cineole at, respectively, 56.7% for *E. globulus* and 25.6% for *E. polybractea*. The main compound in the essential oils of *M. communis* and *R. officinalis* is α-pinene, comprising 43% and 42.5%, respectively, along with a notable amount of 1,8-cineole (28.8% and 9.8%). Citral (neral + geranial) is the main constituent of *C. winterianus* (73.2%) and *L. citriodora* (23.4%) along with limonene (23.1%) for the final oil. The compositions of the other EOs are characterized by various major compounds: *P. asperum* (citronellol 24.5%, geraniol 15.1%, and citronellyl formate 10.5%), *L. hybrida* (linalool 37.3% and linalyl acetate 34.4%), *M. piperita* (menthol 42.7% and menthone 25.2%), *I. graveolens* (bornyl acetate 53% and borneol 19.3%), *P. lentiscus* (α-pinene 25.9% and myrcene 17.5%), and *O. vulgaris* (carvacrol 62% and terpinolene 10.1%).

All these EOs are rich in monoterpenes, primarily oxygenated monoterpenes (alcohols, aldehydes, ketones, ether oxides, and esters) and also monoterpene hydrocarbons like myrcene, limonene, *p*-cymene, and α-pinene. Among the oxygenated monoterpenes, ether oxides are predominantly represented by 1;8-cineole; aldehydes by citral; ketones by menthone; esters by bornyl acetate; and alcohols by linalool, geraniol, menthol, citronellol, and carvacrol, with the latter phenolic compound being a direct result of *p*-cymene hydroxylation [29].

### 3.2. Chemical Composition of Myrtus communis EO and Its Chromatographic Fractions

Based on all previous results and the notable potential of *M. communis* EO to affect the bioluminescence, mobility, and biofilm formation of *A. fischeri*, this EO was selected for further investigation into QS inhibition.

The chemical composition of this essential oil is detailed in Table 2. Forty-one compounds were identified, representing 97.3% of the whole composition. The EO contained mainly monoterpenes, namely α-pinene (43.0%) followed by 1,8-cineole (28.8%), limonene (8.7%), linalool (3.8%), *p*-cymene (2.3%), α-terpineol (1.7%), and geranyl acetate (1.6%), with the other compounds amounting to less than 1% each.

To investigate the anti-*QS* activities of *M. communis* EO, this sample was fractionated to separate molecules based on their polarity. It was submitted to column chromatography (CC) over silica gel using a gradient of solvent pentane/diethyl ether (100/0 to 0/100) and the four fractions (F1–F4) obtained were analyzed by GC(RI) and GS-SM.

The first fraction (F1), eluted with pentane, consists essentially of monoterpene hydrocarbons, mainly α-pinene (70.4%) and limonene (20.1%), which represent 43% and 8.7% of the composition of the whole essential oil, respectively. Fraction F2 (pentane/diethyl ether, 98/2) is composed of 94.2% oxygenated monoterpenes, with 80.8% of 1,8-cineole, the second major component of the EO. F1 and F2 have the highest identification percentages, at 99.2% and 98.3%, respectively. The next two fractions, F3 and F4, which are more complex, are identified at 61.1% and 63.3%.

The F3 fraction (pentane/diethyl ether, 90/10) is the most heterogeneous, containing 41.6% of oxygenated sesquiterpenes, 10.4% of phenylpropanoids (“Others” in Table 2), and 9.1% of oxygenated monoterpenes. The three major compounds of this fraction are caryophyllene oxide (33.5%), methyl eugenol (10.4%), and humulene epoxide II (8.1%), representing 0.3%, 0.2%, and 0.1% of *M. communis* EO, respectively.

F4, eluted with diethyl ether, is composed of 62.5% oxygenated monoterpenes, with small amounts of oxygenated sesquiterpenes (0.2%) and phenylpropanoids (0.6%). The major compounds in this fraction are linalool (27.5%) and α-terpineol (17.6%), two monoterpene alcohols, which account for 3.8% and 1.7% of *M. communis* EO, respectively.

### 3.3. Swimming Assay

**Figure 1:**
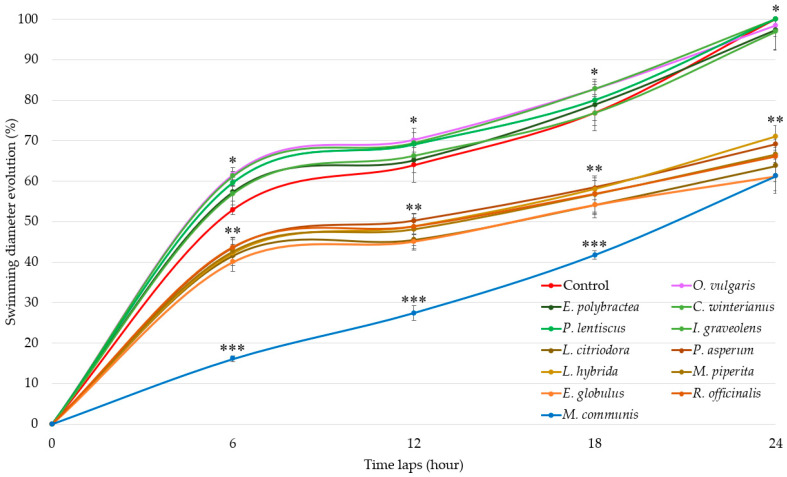
Swimming diameter of *Aliivibrio fischeri* after treatment with or without (control) essential oils during four time laps. EO data are mean values (±SD) of at least four independent measurements (*n* = 3); different (*), (**) and (***) indicate significant differences during each time lap (*p* < 0.001).

Swimming results (Figure 1) show three distinct groups of EOs activities.

EOs of G1 (*E. polybractea*, *C. winteranius*, *I. graveolens*, *P. lentiscus*, and *O. vulgaris*) did not influence *A. fischeri* motility as compared to the control. In contrast, all the other EOs lead to a significant decrease in swimming diameters. The EOs of G2 (*E. globulus*, *R. officinalis*, *L. citriodora*, *P. asperum*, *L. hybrida*, and *M. piperita*) decrease swimming diameter by around 60% at 6 h, 55% at 12 h, 45% at 18 h, and finally by 35% at the end of the 24 h measurement. The most significant delay was observed with *M. communis* EO (blue), which demonstrated stronger motility inhibition starting at 6 h with 36.86% (±0.68%), increasing to 42.94% (±1.80%) at 12 h, then rising further to 54.34% (±1.02%) at 18 h before decreasing to 38.82% (±4.24%) at 24 h, similarly to the G2-active EOs.

### 3.4. Bioluminescence Assay

**Figure 2:**
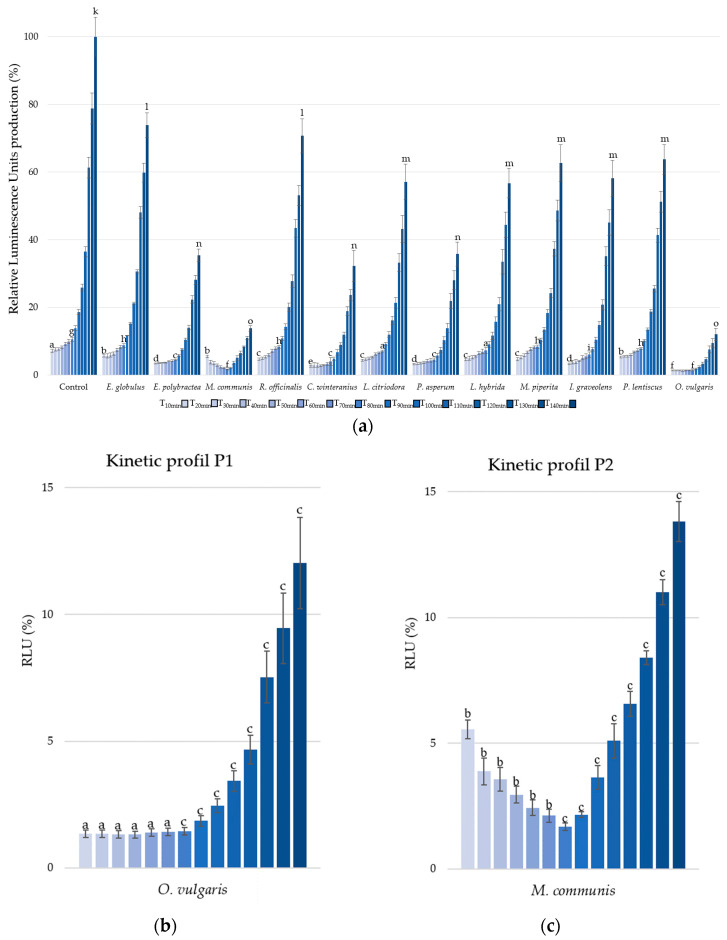
Bioluminescence production of *Aliivibrio fischeri* after treatment with essential oils or without (control). Data are mean values (±SD) of at least four independent measurements (*n* ≥ 4); different lowercase letters indicate significant differences between each EO at three points during the kinetic cycles (1, 7, and 14) (*p* < 0.001) (**a**). Kinetic profiles in bioluminescence inhibition with *O. vulgaris* (**b**) and *M. communis* (**c**) EOs. Data are mean values (±SD) of at least four independent measurements (*n* ≥ 4) different lowercase letters indicate significant differences between the two EOs at each kinetic cycle (*p* < 0.001).

The second parameter, bioluminescence production, was measured under kinetic conditions from T10 minutes to T140 minutes, with 10 min intervals (Figure 2a).

Only three other EOs present significative reduction in RLU, i.e., *E. polybractea*, *C. winteranius*, and *P. asperum* with respective luminescence reduction of 51.98% (±1.39%), 59.91% (±3.62%), and 55.34% (±3.33%) at T10 minutes. At the end of the kinetic cycle, *E. polybractea, C. winteranius*, and *P. asperum* EOs decrease, respectively, RLU by 64.59% (±1.85%) 67.71% (±4.62%), and 64.21% (±3.51%).

But the most significant activities were observed with EOs of *O. vulgaris* and *M. communis*, showing respective luminescence decreases of 86.28% (±0.16%) and 84.17% (±0.15%).

Furthermore, it is important to note that all the EOs and growth controls exhibited similar kinetic profiles, characterized by a stagnation in luminescence followed by an increase, with the sole exception of *M. communis* EO.

Indeed, to compare the kinetic profiles, *O. vulgaris* EO (Figure 2b) was chosen to represent P1 profile, while *M. communis* EO, the only P2 profile, was selected (Figure 2c). Two distinct trends are observed in the *A. fischeri* inhibition of bioluminescence production: the P1-Type, characterized by a stagnation in luminescence production (T1 to T5) before an increase, and the P2-Type, which shows a reduction (T1 to T5) in luminescence production followed by an increase.

### 3.5. Biofilm Assay

**Figure 3:**
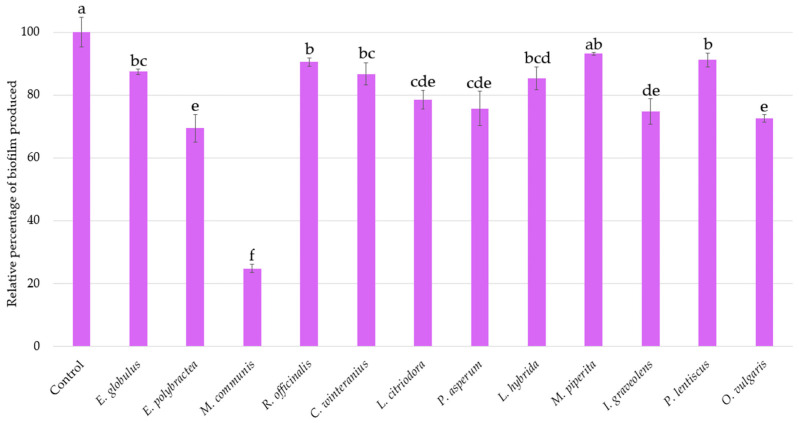
Relative percentages of *Aliivibrio fischeri* biofilm production in the absence (control) or presence of EOs. Data are mean values (±SD) of at least four independent measurements (*n* ≥ 4) different lowercase letters indicate significant differences between each EO (*p* < 0.001).

The same *inoculum* used to assess bioluminescence production was kept for investigating the biofilm production of *A. fischeri* after a 2 h 30 min treatment with each EO (Figure 3). An inhibition of 25% in biofilm formation is considered significant.

The internal control *O. vulgaris* EO reduces biofilm formation by 27% (±1.25%). With similar activities *E. polybractea*, *L. citriodora*, *P. asperum*, and *I. graveolens* EOs decrease biofilm production by 20 to 30%. However, the effect of *M. communis* EO is the most important, with a reduction rate of 75.22% (±1.32%), further highlighting its strong biofilm-reducing activity.

### 3.6. Quorum-Quenching Activity of Myrtus communis EO and Its Chromatographic Fractions

The activities of the four fractions were assessed using the same parameters evaluated for *M. communis* EO: swimming, luminescence, and biofilm production. To select the anti-*QS* concentrations, the MIC of each fraction was determined: F1 and F2 were tested at 50 ppm, while F3 and F4 were used at 25 ppm because of their antibacterial activities at 50 ppm.

**Figure 4:**
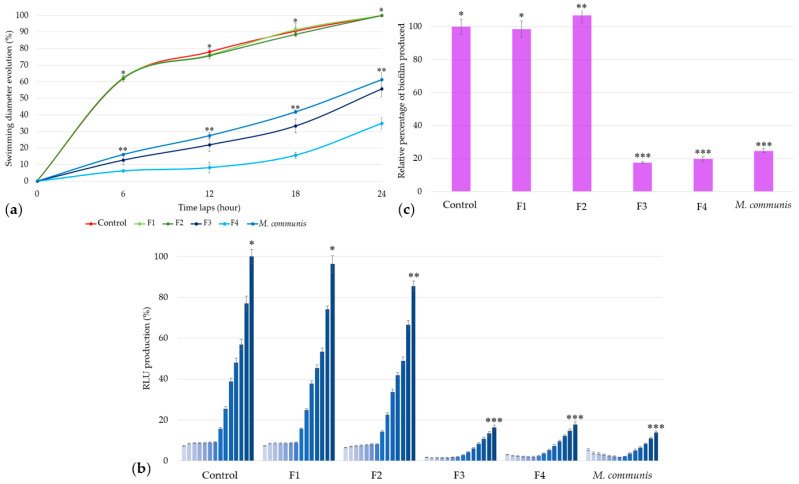
Activity of *M. communis* EO and its chromatographic fractions on *Aliivibrio fischeri* swimming (**a**), bioluminescence (**b**), and biofilm production (**c**). Data are mean values (±SD) of at least four independent measurements (**a**, *n* = 3; **b** and **c**, *n* ≥ 4). Different (*), (**) and (***) indicate significant differences between each EO at each time lap (**a**), at the end of each kinetic cycle (**b**), or at 2 h 30 min (**c**) (*p* < 0.001).

In comparison with results obtained for the bacterial positive control and *M. communis* EO, the F1 and F2 fractions―composed of α-pinene (70.4%) and limonene (20.1%) for the former, and 1,8-cineole (80.8%) for the latter—did not exhibit any activity against *A. fischeri* swimming (Figure 4a), bioluminescence production (Figure 4b), or biofilm formation (Figure 4c).

In contrast to *M. communis* fractions F1 and F2 (G1), the use of 25 ppm of F3 and F4 (G2) against *A. fischeri* resulted in a significant reduction in swimming motility, respectively, 44.31% (±4.75%) and 65.10% (±3.40%) (Figure 4a).

Similarly, bioluminescence production was reduced by 83.68% (±1.23%) and 82.10% (±1.33%) with F3 and F4 at the end of the kinetic cycle. Both fractions show a P2-type profile, with F3 exhibiting a more intense activity at the beginning of the kinetic cycle (Figure 4b).

The relative quantification of biofilm production highlights the effectiveness of F3 and F4, with reductions of 82.44% (±0.66%) and 80.18% (±1.54%), respectively (Figure 4c).

These results show that *M. communis* EO, along with its F3 and F4 extracts, exhibits the strongest activity against *Aliivibrio fischeri* QS, including significant effects on swimming motility, bioluminescence, and biofilm production.

## 4. Discussion

In contrast to data from the literature, chemotype carvacrol *Origanum vulgaris* EO showed significant activity only in disrupting bioluminescence with no notable effect on biofilm formation or swimming [39,40,41]. Conversely, Corsican *Myrtus communis* EO decreased both swimming diameter and the production of bioluminescence as well as biofilm formation. The chemical analysis of *M. communis* EO yielded an identification rate of 97.3%. Among the 41 identified compounds, α-pinene, 1,8-cineole, and limonene were classified as major constituents. However, these main compounds are also present in other essential oils, such as *E. globulus*, *L. citriodora*, *R. officinalis*, and *P. lentiscus*, which do not show notable activity against *quorum-sensing* [42]. To further explore these findings and possibly identify the active molecules, *M. communis* EO was separated into four distinct chromatographic fractions (F1–F4).

Contrary to initial hypotheses, the overall activities of α-pinene, 1,8-cineole, and limonene were not confirmed. The results presented in Figure 4 show that only two fractions of chromatography of *M. communis* EO, F3, and F4, exhibited activity, while F1—which contained all the α-pinene and limonene from *M. communis* EO—did not, contrary to what was previously observed in other Gram-negative *quorum-sensing* studies [43,44]. Neither F1 nor F2, which contained all the 1,8-cineole, showed any activity, in contrast to other strains [45,46]. In contrast and according to the chemical fraction analysis, only the more polar fractions, F3 and F4, exhibited anti-QS activity comparable to that of the *M. communis* EO in all three studied parameters.

**Figure 5:**
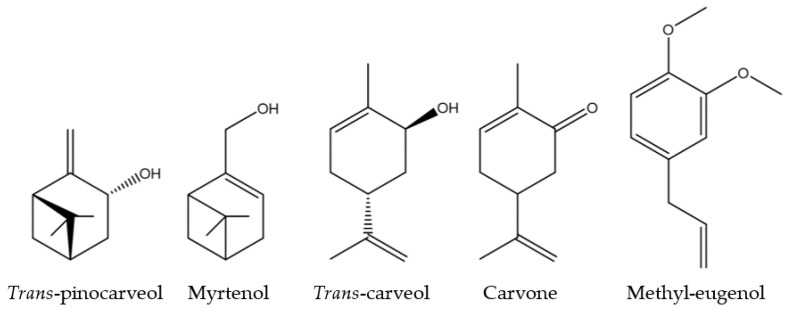
Chemical structure of the five common F3 and F4 fractions molecules.

Both F3 and F4 fractions showed similar activity across the three parameters, mirroring the activity of *M. communis* EO. According to chemical analyses, five molecules, among the identified ones, were found to be common to both F3 and F4: *trans*-pinocarveol, myrtenol, *trans*-carveol, carvone, and methyl-eugenol. All of these molecules are oxygenated monoterpenes, except methyl-eugenol (a phenylpropanoid) (Figure 5).

If the activities of *M. communis* EO can be linked to any of these molecules, it could be due to one of them acting alone, a combination of them, or their interaction with others. All the results suggest that a QS mechanism disruption is involved. Based on previous findings, hydroxyl and carbonyl functional groups seem to be important, as well as the presence of double bonds in cyclic molecules [47,48,49]. *Trans*-pinocarveol and myrtenol are unsaturated bicyclic monoterpenes with a hydroxyl group. *Trans*-carveol and carvone are unsaturated oxygenated monoterpenes with the *p*-menthane skeleton. They have two double bonds (C=C, one cyclic) and are differentiated by a hydroxyl group for *trans*-carveol, replaced by a carbonyl group for carvone. Finally, methyl-eugenol includes an aromatic ring (conjugated planar ring system) substituted by two methoxy groups and an allyl group.

Myrtenol has already demonstrated biofilm reduction ability against *Staphylococcus aureus* [50] and *Klebsiella pneumoniae* associated with antibiotics [51]. Concerning carveol, a study on limonene isomers highlighted that carveol exhibits stronger activity against *Xanthomonas oryzae* compared to limonene [52]. Carveol is a limonene derivative with a hydroxyl group on the C2, which is why the presence of the hydroxyl group is linked to its activity. Another structurally similar molecule to carveol is carvone, which has demonstrated QS disruption in *Chromobacterium violaceum* and *Hafnia alvei*, with a reduction in biofilm formation. Additionally, carvone impairs the motility of these two bacteria [53,54]. Indeed, carvone can reduce acyl-homoserine lactone production by disrupting AHL synthase and the QS transcriptional regulator.

As for methyl-eugenol, several studies have demonstrated its QS disruption abilities in *Chromobacterium violaceum* and *Vibrio harveyi*. This molecule may act as a competitive ligand for CviR, the QS system in *Chromobacterium violaceum* [55,56].

In *Vibrio harveyi*, a reduction in luminescence production has been observed with methyl-eugenol. Among the Gram-negative bacteria mentioned above, the QS systems of *Chromobacterium violaceum* and *Halfnia alvei* are regulated by N-hexanoyl-L-homoserine lactone (C6-HSL), which has a structure similar to N-(3-oxohexanoyl)-L-homoserine lactone (3-oxo-C6-HSL), the QS autoinducer for *Vibrio harveyi* and *Aliivibrio fischeri* [57]. This autoinducer plays a crucial role in bioluminescence production to the LuxR receptor, which initiates transcription. A reduction in 3-oxo-C6-HSL levels could be due to decreased production or inactivation via binding interactions which would directly disrupt biofilm formation and bioluminescence production in *Aliivibrio fischeri*. If *A. fischeri* bioluminescence is majority driven by *Lux*I/R, motility is regulated by *Ain*S/R and *Lux*S/PQ systems [58,59]. This second *Ain*S/R system is regulated by N-octanoyl-homoserine lactone (C8-HSL) also implied in bioluminescence at low cellular density [60].

Therefore, some of these five molecules, either alone or in combination, may interact with 3-oxo-C6-HSL and/or C8-HSL, potentially affecting other bacteria as well. For instance, QS systems of other *Vibrio* species, such as that of *Vibrio anguillarum*, a fish pathogen that can also infect humans, might be impacted [61]. The QS of *Yersinia enterocolitica*, a human pathogen, can also be disrupted because its most produced autoinducer is 3-oxo-C6-HSL [62]. Additionally, *Agrobacterium tumefaciens* and *Erwinia carotovora*, two plant pathogens, could experience reduced tumor production and the diminished activity of enzymes responsible for cell wall degradation due to the inhibition of C8-HSL or 3-oxo-C6-HSL [63,64].

## 5. Conclusions

To conclude, *Myrtus communis* Corsican essential oil is the most active among the twelve essential oils tested in the study. This EO was fractioned by column chromatography and the F3 and F4 oxygenated fractions demonstrated strong *quorum-quenching* activity at a sub-inhibitory concentration by disrupting biofilm formation, mobility, and bioluminescence. The EO of *M. communis* and its F3 and F4 fractions showed stronger activity than the carvacrol-rich *O. vulgaris* EO. Molecules, such as *trans*-pinocarveol, myrtenol, *trans*-carveol, carvone, and methyl eugenol, were identified in both active fractions only. These results highlight the importance of minority compounds which can interfere with QS-related traits. An in-depth study is necessary to elucidate their mechanisms of action and to consider the application of active fractions or compounds against other marine biofilm-forming bacteria with environmental and clinical relevance.

## Figures and Tables

**Table 1:** Major compounds of the eleven Corsican essential oils and *Origanum vulgaris* essential oil (internal control).

EOs	Major Compounds (>5%)
*Eucalyptus globulus*	1,8-cineole 56.7%; α-Pinene 15%; Limonene 5.7%
*Eucalyptus polybractea*	1,8-cineole 25.6%; *p*-Cymene 19%; Spathulenol 6.2%; Crypton 6%
*Myrtus communis*	α-Pinene 43%; 1,8-cineole 28.8%; Limonene 8.7%
*Rosmarinus officinalis*	α-Pinene 42.5%; 1,8-cineole 9.8%; Camphene 8.6%; Bornyl acetate 7.5%
*Cymbopogon winterianus*	Geranial 41.7%; Neral 31.5%; Geraniol 4.8%
*Lippia citriodora*	Limonene 23.1%; Geranial 13.5%; Neral 9.9%; 1,8-cineole 8%; β-Caryophyllene 5.7%; Ar-curcumene 5%
*Pelargonium asperum*	Citronellol 24.5%; Geraniol 15.1%; Citronellyl formate 10.5%; Linalol 6.8%; Isomenthone 6.7%; Geranyl formate 6.1%
*Lavandula hybrida*	Linalol 37.3%; Linalyl acetate 34.4%
*Mentha piperita*	Menthol 42.7%; Menthone 25.2%; 1,8-cineole 6%; Menthyl acetate 5%
*Inula graveolens*	Bornyl acetate 53%; Borneol 19.3%; Camphene 6.4%
*Pistacia lentiscus*	α-Pinene 25.9%; Myrcene 17.5%; α-Phellandrene 6.4%
*Origanum vulgaris*	Carvacrol 62%; Terpinolene 10.1%; γ-Terpinene 8.3%

**Table 2:** Composition of *Myrtus communis* essential oil and the four fractions obtain after chromatography.

No.	Components ^a,b^	RIa^Lit^	RIa BP-1	RIp BP-20	% EO	% F1	% F2	% F3	% F4
1	Isobutyl isobutyrate ^a^	901	900	1094	0.4		0.3		
2	α-Thujene	926	923	1019	0.4	0.6			
3	α-Pinene	934	932	1019	43.0	70.4			
4	Sabinene	968	966	1120	0.1	0.1			
5	β-Pinene	973	971	1114	0.4	0.8			
6	Myrcene	983	981	1163	0.1	0.2			
7	Isobutyl 2-methylbutyrate ^a^	988	988	1178	0.5		1.1		
8	2-Methylbutyl isobutyrate ^a^	1003	1001	1200	0.2		0.5		
9	δ-3-Carene	1007	1006	1151	0.4	0.8			
10	*p*-Cymene	1015	1012	1274	2.3	5.6			
11	Limonene *	1023	1022	1204	8.7	20.1			
12	1,8-Cineole *	1022	1022	1213	28.8		80.8		
13	Linalool oxyde	1065	1059	1442	0.1				0.9
14	Linalool	1086	1087	1544	3.8				27.5
15	2-Methylbutyl 2-methylbutyrate ^a^	1090	1089	1282	0.6		1.8		
16	α-Campholenal	1107	1105	1491	tr		0.8		
17	*Cis*-limonene oxide	1118	1117	1459	0.1		0.3		
18	*Trans*-pinocarveol	1126	1124	1654	0.3			**0.2**	**2.3**
19	*Trans*-verbenol	1133	1129	1677	0.4				3.6
20	Pinocarvone	1140	1139	1568	tr			0.5	
21	*p*-Cymen-8-ol	1164	1160	1847	0.2				2.4
22	Terpinene-4-ol	1164	1162	1601	0.2				1.8
23	Myrtenal	1171	1169	1627	tr			0.7	
24	α-Terpineol	1175	1172	1696	1.7				17.6
25	Estragole ^b^	1175	1174	1687	0.1		0.4		
26	Myrtenol	1182	1179	1792	0.1			**0.2**	**0.7**
27	Verbenone	1184	1182	1702	0.1				1.6
28	*Trans*-carveol	1201	1197	1834	0.1			**0.3**	**1.7**
29	Carvone	1218	1215	1739	0.1			**4.2**	**0.3**
30	Geraniol	1238	1234	1849	0.3				2.1
31	Linalyl acetate	1239	1239	1556	0.7		2.9	1.6	
32	Geranial	1247	1243	1725	0.1			1.4	
33	α-Terpinyl acetate	1333	1332	1696	0.5		2.0		
34	Neryl acetate	1344	1339	1727	0.1		0.3		
35	Geranyl acetate	1361	1359	1758	1.6		7.1		
36	Methyl eugenol	1376	1369	2013	0.2			**10.4**	**0.6**
37	E-β-caryophyllene	1419	1417	1595	0.2	0.4			
38	α-Humulene	1449	1455	1666	tr	0.2			
39	Caryophyllene oxide	1570	1568	1975	0.3			33.5	
40	Humulene epoxide II	1597	1593	2031	0.1			8.1	
41	Epicubenol	1614	1616	2033	tr				0.2
	Monoterpene hydrocarbons				55.4	98.6			
	Oxygenated monoterpenes				39.3		94.2	9.1	62.5
	Sesquiterpene hydrocarbons				0.2	0.6			
	Oxygenated sesquiterpenes				0.4			41.6	0.2
	Others				2.0		4.1	10.4	0.6
	Total				97.3	99.2	98.3	61.1	63.3

Components are listed in order of their elution in apolar column BP-1; percentages in apolar column BP-1, except those with * (% on polar column BP-20); RIa and RIp: retention indices in apolar and polar columns, respectively; RIa^Lit^, the literature retention indices reported in Babushok et al. [34], otherwise written as ^a^ [35] and ^b^ [36]; tr: traces (<0.05%). The molecules common to fractions F3 and F4 are shown in bold.

## Data Availability

The original contributions presented in this study are included in the article. Further inquiries can be directed to the corresponding author.
